# National and transnational belonging among Turkish and Moroccan older migrants in the Netherlands: protective against loneliness?

**DOI:** 10.1007/s10433-017-0420-9

**Published:** 2017-03-30

**Authors:** Jolien Klok, Theo G. van Tilburg, Bianca Suanet, Tineke Fokkema, Martijn Huisman

**Affiliations:** 1 Department of Sociology, Faculty of Social Sciences, Vrije Universiteit Amsterdam, De Boelelaan 1081, 1081 HV Amsterdam, The Netherlands; 2 Netherlands Interdisciplinary Demographic Institute (NIDI-KNAW), Lange Houtstraat 19, 2511 CV Den Haag, The Netherlands; 3 University of Groningen, Broerstraat 1-11, 9712 CP Groningen, The Netherlands; 4 Erasmus University Rotterdam, Burgemeester Oudlaan 50, 3062 PA Rotterdam, The Netherlands; 5 Department of Epidemiology and Biostatistics, EMGO+ Institute for Health and Care Research, VU University Medical Center, De Boelelaan 1089, 1081 HV Amsterdam, The Netherlands

**Keywords:** Transnational belonging, Loneliness, Older migrants, Acculturation strategies, Place attachment

## Abstract

This research investigates how a sense of belonging functions as protective mechanism against loneliness. Inspired by the work of Berry (1980) on acculturation strategies (i.e. integration, assimilation, separation and marginalization), we distinguish migrants who feel a relatively strong or weak sense of belonging to larger society and those who feel a strong or weak belonging to the “own group.” We expect that more national belonging contributes to less loneliness. We add a transnational perspective by arguing that feelings of belonging to the own group can take place in the country of settlement, but can also be transnational, i.e. a feeling of belonging to the country of origin. Transnational belonging can protect against loneliness, as it acknowledges the importance of place attachment. Using data from the Longitudinal Aging Study Amsterdam on older migrants aged 55–66, we employ latent class analysis and find five national belonging clusters, interpretable in terms of Berry’s acculturation strategies. Further analyses reveal mixed evidence: some aspects of transnational belonging vary with belonging to the own group, but other aspects point to a third dimension of belonging. Regression analysis shows that those marginalized are loneliest and that a transnational sense of belonging contributes to more loneliness. We conclude that Berry’s (1980) typology is useful for interpreting older migrants’ national belonging and that a transnational sense of belonging is apparent among older migrants, but needs to be explored further.

## Introduction

Today, many Western countries face two salient phenomena that profoundly change the way societies are organized: population ageing and the globalization of migration (Torres 2013). Consequently, increasing numbers of people age in a foreign land. Due to an accumulation of risk factors, such as low socio-economic position (Reijneveld 1998), poorer health conditions (Denktaş 2011) and facing difficulties that are associated with international migration such as discrimination and social exclusion (Silveira and Allebeck 2001), older migrants are considered socially vulnerable (Cela and Fokkema 2016). Exemplary of this social vulnerability is the finding that older migrants are lonelier than their native peers (Fokkema and Naderi 2013; Victor et al. 2012). Regarding loneliness among this population, there is a specific need to not only study structural factors leading to loneliness, such as low socio-economic position, as has often been done (Victor et al. 2005). Instead, more focus is desired on how belonging from a migrant’s perspective, with all sociocultural precariousness that comes with migration, explains variation in loneliness among older migrants (De Jong Gierveld et al. 2015).

Inspired by Berry’s (1980) model for acculturation strategies, we distinguish between different forms of belonging from a migrant’s perspective. Belonging can take place within the country of settlement, but can also be oriented towards the country of origin, i.e. a transnational belonging. An understanding of how older migrants give meaning to a sense of belonging in an increasingly transnational world is still largely unexplored (Torres 2013). We aim to fill this knowledge gap and study how a sense of belonging functions as protection against loneliness. First, we explore profiles of belonging among older migrants. Second, we determine how these profiles are associated with loneliness. We study Turkish and Moroccan older migrants in the Netherlands, a receiving country for many labour migrants in the 1960s and 1970s. Turkish and Moroccan migrants now form the main migrant groups in the Netherlands, together with Surinamese and Antillean migrants.

### Belonging and loneliness

Departing from loneliness and well-being literature, belonging centres around having social attachments to and interactions with other people (Baumeister and Leary 1995). Elsewhere, belonging is posed as a vital mental health concept and defined in more generic terms: belonging requires a personal involvement in a “system” or “environment” (Hagerty et al. 1992). This is where one can say: “We belong together” or: “I am one of them.” For migrants, belonging is hardly self-evident. After migration ties to family, friends and community in the country of origin are put under pressure (Treas and Batlova 2009). Insecurity about how to socialize and about social expectancies in the new country obstructs the development of a new social network (Watt and Badger 2009). In migration studies as well as in human geography, belonging is brought in relation to a feeling of being “at home” (Yuval-Davis 2006), in which “home” represents a “symbolic space of familiarity, comfort, security and emotional attachment” (Antonsich 2010, p. 650). The significance of home arguably becomes stronger in the context of migration: the sheer fact of being in a different place with different customs and social norms compromises the extent to which one feels a sense of belonging and might lead to uncertainties regarding identity (Lee et al. 2010). Lynd (1958, p. 210) simply put it this way: “Some kind of answer to the question: ‘Where do I belong?’ is necessary for an answer to the question: ‘Who am I?’” A lack of belonging can result in loneliness, deprivation, feeling an outsider and valuing life as unfulfilled and shallow (Verkuyten 2004). Loneliness is a situation experienced by an individual as one where there is a dissatisfying quality or quantity of personal relationships (De Jong Gierveld 1998). This definition concerns the subjective evaluation of actual social relationships and interactions. The notions of belonging mentioned above, tapping into social embeddedness, belonging *somewhere* and being *someone* are combined when we bring “national” and “transnational” belonging in relationship with loneliness in this paper.

Although related, the concepts of belonging and loneliness are not the same (Hagerty et al. 1992). Loneliness is the negative evaluation, or feelings of “missing,” of a particular situation and results from a deficiency to effectuate certain standards regarding social relationships (De Jong Gierveld 1998). Belonging is a description of the social world and not a subjective evaluation. For instance, a person may belong to a certain group (“I am one of them”), but this is not an evaluation in itself, and can thus still experience loneliness. We explain variations in loneliness by studying different forms of belonging (Hagerty and Patusky 1995). We articulate belonging using two components: first, behavioural practice, like social interactions (Anthias 2013) and second, imagined belonging, reflecting being part of a bigger whole, and being at home (Antonsich 2010; Hagerty et al. 1992).

### Belonging to larger society and belonging to the “own group”

Migrants’ sense of belonging can be multifaceted. We use Berry’s work (1980) on acculturation to illustrate some options (although simplified). Acculturation is the process whereby groups with distinctive cultures engage in contact (Berry 2005). Berry (1980) distinguishes four responses to this intercultural contact from a migrant’s perspective, along lines of orientation towards one’s own group and towards the mainstream population. These responses are integration, assimilation, separation and marginalization (Table 1) and contain different implications for well-being (Berry 2005; Sonn 2002): migrants who maintain their own identity and preserve their own culture are generally better off than those who do not (LaFromboise et al. 1993; Phinney et al. 2001).

**Table 1:**
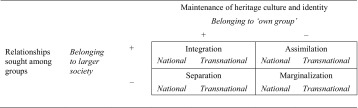
Four acculturation strategies

*Source*: Berry (2005, p. 705); in italic: own addition

We added to this model our own focus of study: a sense of belonging. We draw a parallel on the rows in the model and show in Table 1 that having a strong (+) sense of belonging to the “own group” is corresponding to a relative preference (+) for “maintenance of heritage culture and identity” in Berry’s (1980) model. In the columns, something similar happens. With “relationships sought among groups,” Berry aims to capture an attitude that prefers to actively seek contact with, and participate in larger society, which also encompasses “other” (minority) ethno-cultural groups. Again, we draw a parallel: relative preference (+) for engagement in larger society is analogous to having a strong (+) sense of belonging to larger society. In our model, the outcomes shown in Table 1 do not refer to a position in society where one is (un)able to participate economically or socially, or where adaption of cultural norms and values takes place (Berry 2005). They are, instead, an indication of an individual experience of being socially embedded (behaviour) and being at home (imagination), whatever the position in society. Thus, “integration” in Berry’s terminology, i.e. participating in society, in our model means a feeling of belonging to the own group as well as to larger society.

A sense of belonging is associated with less loneliness (De Jong Gierveld et al. 2015; Prieto-Flores et al. 2011). Hence, we propose that marginalized older migrants (those belonging nowhere) are loneliest. Older migrants who have a separated and assimilated sense of belonging are expected to be less lonely than older migrants who face marginalized belonging, because both have a strong belonging towards either larger society or the own group. Least lonely, we argue, are those integrated, because of their strong belonging to larger society as well as to the own group. Hypothesis 1 is: older migrants with a marginalized belonging experience the most loneliness, followed by those with assimilated and separated belonging, whereas older migrants with an integrated belonging are least lonely.

### Transnational belonging

Berry (2005) focuses on acculturation *within* the country of settlement. In our model, a sense of belonging to one’s own group could be directed to both the own group *in the country of settlement* (Table 1; national belonging) and the own group *in the country of origin* (transnational belonging).

The complex whole of affiliations and connections that migrants employ during their lives, thereby linking their societies of origin and settlement, was termed “transnationalism” (Glick Schiller, Basch and Blanc-Szanton 1992). In the field of transnationalism, behaviour is distinguished from imagination. There, other terminology is prevalent. “Ways of being” (behaviour) is referred to as actual border-crossing behaviour and social relations. “Ways of belonging” (imagination) signals an identity component: the transnational way of life is a central element of the self, through memory, imagination and nostalgia (Levitt and Glick Schiller 2004). We do not follow this terminology, but differentiate between transnational belonging as behaviour and as imagination. Combining the two to indicate a transnational orientation is not new to the field of transnationalism (Boccagni 2012b; Levitt and Glick Schiller 2004). We argue that (a lack of) belonging *within* country borders, as we have discussed using Berry’s (1980) acculturation model, is complemented by another dimension: transnational belonging. Transnational belonging *crosses* borders between countries of origin and settlement, behaviourally or imaginary.

Transnational belonging acknowledges the importance of place and could therefore play a decisive role in explaining loneliness. Surely, being in the country of settlement and immersing in the own group within this country is not the same as travelling back and forth *between* both countries, keeping in touch with people *in* the country of origin and being involved in that *place* of origin. Place attachment has caught scientific interest and refers to peoples’ attachment to physical locations (Gustafson 2001; Rubinstein and Parmelee 1992). Place attachment increases with old age and gains importance for its impact on well-being in older age (Buffel 2015; Wiles et al. 2009). Homesickness is a common reaction to geographical relocation and captures missing social bonds, but also missing places (Vingerhoets 2005). Therefore, although people may have migrated together with their spouse and children and reside among other migrants from the same community, homesickness for childhood neighbourhoods or countries of birth still occurs (Baldassar 2008). For first-generation older migrants, this “longing for a place” is significant. They were born and raised in a different place and vivid memories thereof leading to nostalgia for the old country in its social and physical sense (Baldassar 2008). Many travel back and forth on a regular basis (Baykara-Krumme 2013). Moreover, approaching retirement age, some consider returning to the country of origin permanently (De Haas and Fokkema 2010), possibly reinforcing place attachment. A sense of belonging to the own group within the country of settlement will probably not obviate feelings of homesickness for a place. Visiting the country of origin, either behaviourally of imaginary, might be more able to fulfil this yearning for a locality or could prove to have a protective function against the psychological ill effects that acculturation poses.

To resume, we propose that next to belonging *within* the country of settlement, a transnational sense of belonging is a dimension of belonging, which does not blindly go along with the extent to which one feels to belong to the own group in the country of settlement or the larger society of settlement. As for other forms of belonging, we expect that transnational belonging protects against loneliness. This leads to Hypothesis 2: A transnational belonging has a protective effect on loneliness above belonging to larger society in the country of settlement and the own group in the country of settlement.

## Methods

### Sample

Data were obtained from an older migrant sample of the Longitudinal Aging Study Amsterdam (LASA; Huisman et al. 2011). The survey was held in 2013 and 2014 and is based on a sample of Turkish (*N* = 269) and Moroccan (*N* = 209) migrants born between 1948 and 1957. The majority of older migrants live in large cities, and therefore, municipal registers of 15 Dutch cities with population sizes ranging from 85 to 805 thousand inhabitants provided the sampling frame. Face-to-face interviews were conducted by trained interviewees who offered a Dutch and translated interview (in Turkish, Moroccan Arabic/Darija and Tarafit). The cooperation rate was 45%.

We exclude three respondents who were institutionalized, five who were not born in either Turkey or Morocco because we are interested in first-generation migrants from these countries, and nine respondents with a premature termination of the interview, leaving a sample of *N* = 461. On average, respondents had spent 36.8 years in the Netherlands and their age ranged from 55 to 66, with a mean of 60.9.

### Measurements

*Loneliness* is measured by an 11-item scale (De Jong Gierveld and Van Tilburg 1999). Exemplary items are: “I miss a really good friend” and “There are plenty of people I can lean on when I have problems.” Scale values ranged from 0 to 11 (*M* = 5.19); reliability is .83.

For national belonging we measure a feeling of belonging to larger society by three variables indicating behaviour and two indicating imagination. We inquired about *Dutch language proficiency*, as this is a symbol of group belonging (Vedder and Virta 2005). We use three items (Kleijn and Verboom 2004), e.g. “I can understand spoken Dutch well.” Response categories ranged from “strongly disagree” (1) to “strongly agree” (4). Scores are summed; reliability is .84. Participation in *social organizations* provides the context for a sense of belonging and meaningful social engagement (Sonn 2002). We asked whether respondents were active in social organizations, such as interest groups. If so, we asked whether there were many Dutch members in the organization. We distinguished “not active, or only in organizations with few or no Dutch people” (0) and “active in one or more organizations with predominantly or many Dutch people” (1). The relationship between contact with people from the mainstream population and a sense of belonging to the country of settlement was postulated by De Jong Gierveld et al. (2015). *Contact frequency with Dutch nonkin* (De Graaf et al. 2010) contains two items, e.g. “How often do you have contact with Dutch or “other” neighbours?” Answer categories ranged from “few times a year or less” (1) to “every day” (4). We use the mean score. *Cultural distance* inquired about the extent to which older migrants allow citizens of the society of settlement in their personal spheres and thus touches upon notions of belonging (Antonsich 2010). Three items were presented, e.g. “I would like to speak to Dutch acquaintances about what worries me” (Kleijn and Verboom 2004). Response categories ranged from “strongly agree” (1) to “strongly disagree” (4). Scores are summed; reliability is .73. *Self*-*identification* was asked as “To which ethnic group do you consider yourself to belong?” Responses were: “Dutch,” “Turkish,” “Kurdish,” “Moroccan Arabic,” “Moroccan Berber” and “other.” In the last category 33 respondents identified themselves as “Turkish/Moroccan and Dutch.” This response and the response “Dutch” are taken as an indication of belonging to larger society (Stronks et al. 2009).

A second dimension of national belonging is to the own group; two variables indicate behaviour and two imagination. With regard to religious identities, Ehrkamp (2007) argues that communal places offer a sense of community, home and belonging to its members. We thus take into account frequency of *mosque attendance*. Response categories ranged from “never” (1) to “once per week or more often” (6). Having contact with neighbours from the same country of origin enhances national belonging (Buffel et al. 2013)*. Contact frequency with Turkish/Moroccan nonkin* (De Graaf et al. 2010) is assessed similarly as described above. *Cultural identity* indicates a sense of belonging to a group or culture (Van Oudenhoven et al. 2006) and consists of a four-item scale. It measures how often respondents participated in activities in conjunction with their own group or their own language, thereby expressing cultural identity, values, attitudes and abilities (Yamada et al. 1997). The scale specifically focuses on activities in the Netherlands. An example is: “Talking to or discuss what’s new with others from the Turkish/Moroccan group.” Response categories were “never” (1) to “often” (4). Scores are summed; reliability is .67. Lastly, the *self*-*identification* variable, response categories are “Turkish, Kurdish, Moroccan or Berber.” As the self-identification question did not specify a locality, but only asked for ethnic group, we did not take this response option as an indicator for transnational belonging.

Transnational belonging is grasped by four variables measuring behaviour and two measuring imagination. The country of origin plays a prominent role in ones’ life when it hosts close family members (Burholt et al. 2016). Hence, frequent contact with family members living in Turkey/Morocco indicates transnational belonging. *Frequent contact with children in Turkey/Morocco* combines contact frequency with children and residence of children. We distinguish between “weekly or daily contact with children in Turkey/Morocco” (1) versus “all other frequencies of contact and residence of children” (0). We follow the same procedure for *frequent contact with extended family in Turkey/Morocco*. Visiting the country of origin is an obvious indicator (Duval 2004). Therefore, *visiting frequency Turkey/Morocco* assessed whether respondents had “not visited in the last 5 years” (1), “had visited in the last 5 years” (2) or “visited in the last year” (3). Actively seeking medical care in Turkey/Morocco, instead of making use of care provisions in the country of settlement, reveals attachment to the country of origin and a sense of “home” associated with it (Lee et al. 2010). Hence, we assessed if respondents recently received dispensable *medical care* in Turkey/Morocco (1), versus not or receiving help because one got sick while being on holiday (0). *Considering return migration* asked whether respondents were considering going back to the country of origin permanently, an issue particularly relevant for older migrants (Hunter 2011) and an indicator of belonging to the country of origin (Ganga 2006). We distinguish between “no” or “I do not know” (0) and “yes” (1). We presented five statements related to *feelings of loss* with regard to the country of origin and hence disclosed attachment to it, e.g. “I belong here less than in Turkey/Morocco.” Scores for not (0) or applicable (1) are summed; reliability is .70.

We take into account demographic characteristics associated with loneliness. Loneliness is higher among people that are older, which has often been explained by the loss of age peers and incapacity of older adults and their network members to participate in social activities (Pinquart and Sörensen 2001). We thus control for *age*. Previous research also established that loneliness is higher among women, which has been linked to socialization processes and less opportunities for women to maintain nonkin ties due to care roles, leading us to control for gender; *female* (Pinquart and Sörensen 2001). Loneliness was also found to be higher among those with low socio-economic status as people with higher socio-economic status tend to have more personal and social resources to maintain social relationships (Pinquart and Sörensen 2001). Therefore, we control for *level of education* (low, middle and high level; values 1–3; *M* = 1.3) and employment status, indicated by *having a paid job* (24%) or not. We distinguish between *married* (78%) versus not married (primarily widowed), as those that are not married are less likely to have an intimate attachment (De Jong Gierveld 1998). Poor health is also related to loneliness, since poor health can limit mobility and hence hinder maintaining social ties (e.g. De Jong Gierveld 1998). *Physical functioning* reflects the ability to perform seven activities of daily living (ADL; Katz et al. 1963); response options are “no, cannot” (0) to “yes, without help” (4). Sum scores are used (*M* = 23.4); reliability is .84. For *self*-*rated health* we asked: “How is your health in general?” with response options “poor” (0) to “excellent” (4) (*M* = 1.5). *Length of residence in the Netherlands* (*M* = 36.8 years) is accounted for, because those that lived in the country of settlement for a longer period of time experience less loneliness, since they have had more time to build up social networks there (Neto 2002). Lastly, we controlled for being of Turkish (56%) or *Moroccan* descent.

### Procedure

First, we employ latent class analysis in Mplus to group respondents who have a high similarity in scores on variables for national belonging, i.e. belonging to larger society and the own group. We expect to find clusters coined by Berry (2005) as marginalized, separated, assimilated and integrated. Comparing the mean score on every variable for each cluster determines the labelling. A second cluster analysis shows that transnational belonging variables do not converge into distinct clusters. The last step is regression analysis to determine which cluster is least lonely (Model 1) and if and how different indicators of transnational belonging impact loneliness (Model 2). The final Model 3 includes control variables.

## Results

### National belonging

In the latent class analysis, five clusters are the best fitting solution, which are interpretable in terms of Berry’s (1980) categorization; the fifth shows similarities with two strategies. Table 2 shows the cluster means for each variable, by acculturation category.

**Table 2:** Sample and cluster means per belonging variable, by acculturation strategy

	All (*N* = 461)		Marginalization (*N* = 76)	Marginalization/separation (*N* = 97)	Integration (*N* = 134)	Assimilation (*N* = 78)	Separation (*N* = 76)	(*F*/*χ* ^2^)
	*M*	*SD*	*M*	*M*	*M*	*M*	*M*	
*National belonging: to larger society*								
Behaviour								
Dutch proficiency (3–12)	7.40	2.40	6.22	6.54	8.88	8.77	5.66	53.4***
Social organizations (0–1)	0.27	0.45	0.17	0.18	0.39	0.56	0.00	23.7***
Contact frequency with Dutch nonkin (1–4)	2.42	0.94	1.49	1.51	3.11	2.89	2.80	160.9***
Imagination								
Cultural distance (3–12)	7.59	2.50	8.91	8.79	6.15	5.57	9.33	70.0***
Self-identification Turkish/Moroccan and Dutch (0–1)	0.07	0.26	0.09	0.07	0.07	0.12	0.00	2.2
Self-identification Dutch (0–1)	0.12	0.33	0.16	0.06	0.14	0.26	0.00	7.4***
*National belonging: to own group*								
Behaviour								
Mosque attendance (1–6)	4.48	1.96	1.86	5.73	5.76	1.92	5.84	771.8***
Contact frequency with Turkish/Moroccan nonkin (1–4)	2.77	0.86	2.14	2.35	3.13	2.53	3.53	56.7***
Imagination								
Cultural identity (4–16)	8.55	2.62	7.14	7.20	9.50	8.91	9.67	24.4***
Self-identification Turkish, Kurdish, Moroccan or Berber (0–1)	0.77	0.42	0.74	0.82	0.75	0.54	1.00	13.4***
*Transnational belonging*								
Behaviour								
Frequent contact with children in Turkey/Morocco (0–1)	0.07	0.25	0.11	0.07	0.04	0.08	0.05	0.8
Frequent contact with extended family in Turkey/Morocco (0–1)	0.21	0.41	0.17	0.18	0.26	0.23	0.17	1.1
Visiting frequency Turkey/Morocco (1–3)	1.73	0.80	1.72	1.81	1.69	1.46	1.99	4.6**
Medical care in Turkey/Morocco (0–1)	0.10	0.29	0.07	0.13	0.10	0.06	0.11	0.9
Imagination								
Considering return migration (0–1)	0.26	0.44	0.21	0.32	0.29	0.19	0.25	1.3
Feelings of loss (0–5)	3.40	1.49	3.41	3.55	3.48	2.72	3.72	5.5***

Variables for national belonging were used to compose the five clusters

* *p* < .05; ** *p* < .01; *** *p* < .001

In the marginalization cluster scores indicate low belonging to one’s own group, but also low belonging to larger society. For instance, participation in activities in conjunction with the own language or cultural group is lowest and comprehension of Dutch is poor. In addition, frequency of contact with Turkish/Moroccan, as well as with Dutch nonkin, is lowest. One cluster, which we termed marginalization/separation, shows most similarity with the marginalized cluster, but with a little extra focus on the own group. Only mosque attendance differs substantially, with the marginalized/separated cluster indicating a higher score. Also, respondents in the last mentioned cluster identify themselves as Dutch to a lesser extent than the marginalized cluster.

The integration cluster contains average scores on many variables as well, but is characterized by a relatively strong sense of belonging to the own group (mosque attendance and cultural identity levels are high), as well as to larger society. Dutch proficiency is high, cultural distance to Dutch larger society is low, and there is frequent contact with Dutch nonkin, as well as with nonkin in the own group. Scores in the assimilation cluster are indicative of a sense of belonging to larger society. Scores are highest on the self-identification dummies that indicate belonging to Dutch larger society and mosque attendance is low. Further, cultural distance is lowest and participation in organizations with many Dutch members highest. The separation cluster conveys strong belonging to the own group. All respondents identify themselves as Turkish, Kurdish, Moroccan or Berber. They go to the mosque the most and participate frequently in activities in conjunction with the own language or cultural group. The separation cluster experiences great cultural distance and has most contact with Turkish/Moroccan nonkin, in comparison to the other clusters.

### Transnational belonging

Next, we employ analysis of variance on the indicators for transnational belonging (Table 2). The five clusters differ in their visiting frequency to Turkey/Morocco and their feelings of loss. Those in the separation cluster have the highest frequency of visiting the country of origin, followed by those in the marginalization/separation, the marginalization, the integration and the assimilated cluster. For feelings of loss a similar pattern was observed. Thus, two of the six indicators are not independent from Berry’s categorization, but four are. Consequently, the results are not unambiguous with respect to whether or not transnational belonging is a different dimension of belonging. Because correlations between the six aspects are low (|r| ranges between .01 and .12), we additionally suggest the indicators are not unequivocally measuring transnational belonging as one concept.

### National and transnational belonging and loneliness

In the regression of loneliness, the separation cluster functions as reference category for the clusters of belonging (Table 3). Supporting Hypothesis 1, the results from this analysis and subsequent analyses with other categories of reference (results not shown) reveal that the marginalization and the marginalization/separation clusters are lonelier than the integration, assimilation and separation clusters; there are no differences among the two and three clusters, respectively.

**Table 3:** Regression of loneliness on national belonging clusters, transnational belonging and control variables (*N* = 461)

	Model 1		Model 2		Model 3	
	*B*	SE	*B*	SE	*B*	SE
Constant	4.54	0.36***	3.47	0.63***	12.64	3.27***
Marginalization (vs. separation)	1.54	0.51**	1.63	0.51**	1.16	0.52*
Marginalization/separation (vs. separation)	1.81	0.49***	1.82	0.48***	1.36	0.47**
Integration (vs. separation)	−0.02	0.46	0.04	0.46	−0.01	0.44
Assimilation (vs. separation)	0.13	0.51	0.46	0.53	0.42	0.53
Frequent contact with children in Turkey/Morocco (0–1)			−0.06	0.59	−0.32	0.57
Frequent contact with extended family in Turkey/Morocco (0–1)			−0.42	0.37	−0.26	0.35
Visiting frequency Turkey/Morocco (1–3)			−0.13	0.19	−0.26	0.18
Medical care in Turkey/Morocco (0–1)			−0.01	0.50	−0.11	0.48
Considering return migration (0–1)			0.43	0.34	0.33	0.33
Feelings of loss (0–5)			0.35	0.10***	0.24	0.10*
Age (55–66)					−0.01	0.05
Female (vs. male)					−1.02	0.33**
Level of education (1–3)					−0.60	0.25*
Having a paid job (vs. no paid job)					−0.29	0.36
Married (vs. not married)					−1.13	0.36**
Physical functioning (0–28)					−0.05	0.03
Self-rated health (0–4)					−0.57	0.15***
Moroccan (vs. Turkish)					−0.87	0.31**
Length of residence in the Netherlands					0.00	0.02
*R* ^2^	0.06		0.09		0.22	
*F* change	7.6***		2.6*		7.8***	

* *p* < .05; ** *p* < .01; *** *p* < .001

In Model 2, we add variables for transnational belonging. Associations for the clusters do not change substantially. Of the transnational variables, only feelings of loss are independently associated with loneliness. A higher level of transnational belonging (as it is embodied by feelings of loss) contributes to more loneliness, as we propose in Hypothesis 2. Visiting Turkey/Morocco, which significantly differed between the clusters (Table 2), is not associated with loneliness.

Model 3 brings control variables to the fore. Still the marginalized marginalized/separated remain lonelier than the separated, although the difference is smaller compared to Models 1 and 2. Feelings of loss also continue to be associated with more loneliness. Being born in Morocco decreases the likelihood of reporting loneliness compared to being born in Turkey. The same goes for being female, being married, having a higher education and having better self-rated health.

## Discussion

In this study, we investigated whether migrants’ belonging impacts loneliness. We studied three dimensions of belonging: to larger society and to the “own group” in the country of settlement, and transnational belonging. Latent class analysis on the first two dimensions of belonging identified five clusters: marginalized, marginalized/separated, integrated, assimilated and separated migrants. We found evidence that transnational belonging is a separate dimension of belonging but results also indicate that it is partly an extension of belonging to the own group in the country of settlement. Hence, we conclude that we have not unravelled whether transnational belonging is a different dimension of belonging.

We found some support for Hypothesis 1: migrants in the marginalization and marginalization/separation cluster are lonelier than migrants in other clusters that have a stronger sense of belonging, i.e. integration, assimilation and separation. Yet, the integration cluster did not show less loneliness than the assimilation or separation clusters. This means that some form of belonging to a certain group is important as a protection against loneliness, but that this does not necessarily has to involve the larger society of settlement. A strong sense of belonging to the own group (separation) is just as effective against loneliness as is integration (or assimilation).

Hypothesis 2 proposed that transnational belonging also functions as a protective mechanism against loneliness. We did not find support for this hypothesis. Where visiting frequency of Turkey/Morocco played a role in distinguishing different levels of transnational belonging between the clusters, it did not decrease loneliness. Even more so, the only significant effect we found for indicators of transnational belonging pertains to feelings of loss and was directed oppositely: more transnational belonging increases loneliness. We further find that the additional effect of personal socio-demographic resources reduces the risk of loneliness, which has been found in studies among nonmigrants (De Jong Gierveld 1998).

That transnational belonging does not decrease loneliness might be explained theoretically. Scholars have argued that being ingrained in two places makes for “betwixt and between” identities (Grillo 2007), or “double absence” (Sayad 1999). Where the relation between nearby social relationships and loneliness is rather obvious, the same apparently does not hold for distant ones. In previous research on ICT-based “co-presence” in transnational relationships, it was concluded that the golden standard remains to be face-to-face social contact (Baldassar 2008). This could mean that a transnational lifestyle contributes to homesickness and loneliness, instead of alleviating it, and causes “uprootedness,” i.e. a diminished sense of belonging.

There appears to be a second explanation for why feelings of loss result in more loneliness. Even though the variable feelings of loss captured an orientation towards the country of origin conceptually, the scale contained negatively presented items that were closely related to the concept of loneliness, such as “I miss people…” Feelings of loss were expected to be telling of transnational belonging in combination with the other variables (such as visiting the country of origin), but by itself it may have tapped into loneliness.

This brings up some methodological issues. Against the backdrop of a lack of large-scale survey data among older migrant populations (Fokkema and Naderi 2013), the LASA data provided us with a unique opportunity to systematically study older migrants’ loneliness. Nevertheless, innate to secondary data analysis as we have employed here, this has also brought some limitations regarding the relation between variables and concepts. For instance, perhaps our measurements of transnational belonging are not exhaustively grasping the concept. However, extensive research on this topic has not resulted in true consensus or clear articulation of what transnational belonging exactly entails, let alone offered unambiguous operationalization of the concept (Boccagni 2012a). Moreover, the data available did adhere to many of the elements that were mentioned in the literature in relation to transnational belonging, as described in the measurements section. Framed into the body of knowledge on explaining differences in loneliness, distinguishing an interlinked behavioural and imagined component in belonging (Hagerty and Patusky 1995; Levitt and Glick Schiller 2004) was helpful.

Further, we relied on cross-sectional data, which implies that the findings in this study are only a snapshot of reality. Belonging to a group of people, to a place and to a plethora of other social categories is not a stable feature over time (Yuval-Davis 2006). This means that the dimensions of belonging are not “end states” or ultimate destinations, nor do they relate to each other steadily over time. It further means that we were not able to test causal mechanisms. We interpreted the results congruent with the theoretical foundation for belonging to lead to loneliness (Baumeister and Leary 1995) instead of the reversed.

The acculturation model, gaining wide popularity in social science as well as in the public debate since Berry’s (1980) use of it, has been widely critiqued (Rudmin 2003). It was put forward as too simplistic (Schwartz and Zamboanga 2008), as presenting an erroneously static and homogenous interpretation of culture with the fourfold categories opted by Berry as end states of a linear process (Hermans and Kempen 1998), thereby leaving no room for the dynamics and complexity of human development, nor for its interaction with context (Bhatia and Ram 2009). Despite valid critiques, the acculturation model succeeds to identify factors that are prominent in migrants’ experiences (Burholt 2004). Especially when explaining loneliness among older migrants, there have been repeated calls to not only study migrant’s vulnerability for loneliness (as a group), but to look specifically at differences—among others, in sociocultural embeddedness—within this group and thus explain how loneliness varies among migrants (De Jong Gierveld et al. 2015; Fokkema and Naderi 2013; Wu and Penning 2013). Moreover, we did not aim to present larger society and the own group as socially cohesive or homogenous. We approach the model from the viewpoint of individuals finding a sense of belonging and a “home” in society, rather than adapting to a specific set of cultural norms. The fact that we find groups interpretable of Berry’s (2005) categorization also suggests that its merit in reflecting social reality is still tenable.

In the Netherlands, Turkish and Moroccan migrants have experienced conditions that are commonly found among migrant populations in Europe and beyond: on average they have a lower socio-economic position and worse health than the native population. Due to this dominant pattern of disadvantage and their similar migration backgrounds as guest workers and subsequent family reunifications, we did not study Turkish and Moroccan migrants separately (Denktaş 2011). The focus on people with a migratory past for whom a sense of belonging is not obvious applies to both migrant groups. Moreover, both are known to have strong ties with their country of origin (Fokkema et al. 2016). However, it might be the case that a sense of belonging to larger society and to the own group, as well as a transnational belonging differs between Turkish and Moroccan migrants, which would obscure our results. For example, we were left with the finding that Turkish migrants are lonelier than Moroccan migrants, which was similarly observed in a previous Dutch study (Uysal-Bozkir et al. 2015).

Despite these limitations, this study has various innovations. We have connected a theory on loneliness provoking factors with theoretical insights on migrants’ social integration. Therefore, it provides new insights on variations in loneliness among migrants, which has repeatedly been called for. Second, we added a transnational lens, which, until recently, has mostly been neglected in research on ageing (Torres 2013). Third, we empirically added a transnational dimension to a two-dimensional acculturation model, something that was previously suggested by Van Oudenhoven et al. (2006). Although theorizations and qualitative research on transnational orientations are manifold, we find mixed evidence for transnational belonging as a separate dimension of belonging and that this was not vital for understanding differences in loneliness. Yet, transnational belonging is apparent for all forms of belonging distinguished in this paper. Consequently, more attention and prominence should be given to transnational belonging in empirical research among older migrants in general, and further understanding on how it relates to well-being specifically.

## References

[CR1] Anthias F (2013). Moving beyond the Janus face of integration and diversity discourses: towards an intersectional framing. Sociol Rev.

[CR2] Antonsich M (2010). Searching for belonging—an analytical framework. Geogr Compass.

[CR3] Baldassar L (2008). Missing kin and longing to be together: emotions and the construction of co-presence in transnational relationships. J Intercult Stud.

[CR4] Baumeister R, Leary M (1995). The need to belong: desire for interpersonal attachments as a fundamental human motivation. Psychol Bull.

[CR5] Baykara-Krumme H (2013). Returning, staying, or both? Mobility patterns among elderly Turkish migrants after retirement. Transnatl Soc Rev.

[CR6] Berry JW, Padilla A (1980). Acculturation as varieties of adaptation. Acculturation: theory, models and findings.

[CR7] Berry JW (2005). Acculturation: living successfully in two cultures. Int J Intercult Relat.

[CR8] Bhatia S, Ram A (2009). Theorizing identity in transnational and diaspora cultures: a critical approach to acculturation. Int J Intercult Relat.

[CR9] Boccagni P, Vargas-Silva C (2012). Even a transnational social field must have its boundaries: Methodological options, potentials and dilemmas for researching transnationalism. Handbook of research methods in migration.

[CR10] Boccagni P (2012). Rethinking transnational studies: transnational ties and the transnationalism of everyday life. Eur J Soc Theory.

[CR11] Buffel T (2015). Ageing migrants and the creation of home: mobility and the maintenance of transnational ties. Popul Space Place.

[CR12] Buffel T, Phillipson C, Scharf T (2013). Experiences of neighbourhood exclusion and inclusion among older people living in deprived inner-city areas in Belgium and England. Ageing Soc.

[CR13] Burholt V (2004). The settlement patterns and residential histories of older Gujaratis, Punjabis and Sylhetis in Birmingham, England. Ageing Soc.

[CR14] Burholt V, Dobbs C, Victor C (2016). Transnational relationships and cultural identity of older migrants. GeroPsych.

[CR15] Cela E, Fokkema T (2016). Being lonely later in life: a qualitative study among Albanians and Moroccans in Italy. Ageing Soc.

[CR16] De Graaf PM, Kalmijn M, Kraaykamp G, Monden CWS (2010) Design and content of the NEtherlands Longitudinal Lifecourse Study (NELLS). Research report, Tilburg University and Radboud University Nijmegen, Netherlands

[CR17] De Haas H, Fokkema T (2010). Intra-household conflicts in migration decision making: return and pendulum migration in Morocco. Popul Dev Rev.

[CR18] De Jong Gierveld J (1998). A review of loneliness: concept and definitions, determinants and consequences. Rev Clin Gerontol.

[CR19] De Jong Gierveld J, Van Tilburg TG (1999). Manual of the loneliness scale.

[CR20] De Jong Gierveld J, Van der Pas S, Keating N (2015). Loneliness of older immigrant groups in Canada: effects of ethnic-cultural background. J Cross Cult Gerontol.

[CR21] Denktaş S (2011) Health and health care use of elderly immigrants in the Netherlands: a comparative study. Dissertation, Erasmus University Medical Center, Rotterdam

[CR22] Duval DT (2004). Linking return visits and return migration among Commonwealth Eastern Caribbean migrants in Toronto. Glob Netw.

[CR23] Ehrkamp P (2007) Beyond the mosque: Turkish immigrants and the practice and politics of Islam in Duisburg-Marxloh, Germany. In: Aitchison C, Hopkins P, Kwan M (eds) Geographies of muslim identities: diaspora, gender and belonging Aldershot, Ashgate, pp 11–28

[CR24] Fokkema T, Naderi R (2013). Differences in late-life loneliness: a comparison between Turkish and native-born older adults in Germany. Eur J Ageing.

[CR25] Fokkema T, Cela E, Witter Y, Horn V, Schweppe C (2016). Pendular migration of the older first generations in Europe: misconceptions and nuances. Transnational aging. Current insights and future challenges.

[CR26] Ganga D (2006). From potential returnees into settlers: Nottingham’s older Italians. J Ethn Migr Stud.

[CR27] Glick Schiller N, Basch L, Blanc-Szanton C (1992). Towards a definition of transnationalism. Introductory remarks and research questions. Ann N Y Acad Sci.

[CR28] Grillo R (2007). Betwixt and between: trajectories and projects of transmigration. J Ethn Migr Stud.

[CR29] Gustafson P (2001). Roots and routes: exploring the relationship between place attachment and mobility. Environ Behav.

[CR30] Hagerty BM, Patusky K (1995). Developing a measure of sense of belonging. Nurs Res.

[CR31] Hagerty BM, Lynch-Sauer J, Patusky KL, Bouwsema M, Collier P (1992). Sense of belonging: a vital mental health concept. Arch Psychiatr Nurs.

[CR32] Hermans HJM, Kempen HJG (1998). Moving cultures. The perilous problems of cultural dichotomies in a globalizing society. Am Psychol.

[CR33] Huisman M, Poppelaars J, Van der Horst M, Beekman AT, Brug J, Van Tilburg TG, Deeg DJ (2011). Cohort profile: the longitudinal aging study Amsterdam. Int J Epidemiol.

[CR34] Hunter A (2011). Theory and practice of return migration at retirement: the case of migrant worker hostel residents in France. Popul Space Place.

[CR35] Katz S, Ford AB, Moskowitz RW, Jackson BA, Jaffe MW (1963). Studies of illness in the aged. The index of ADL: a standardized measure of biological and psychological function. J Am Med Assoc.

[CR36] Kleijn WC, Verboom R (2004) Taal en Cultuur Index (TECI). Centrum ‘45, Altrecht, Universiteit Leiden, Netherlands

[CR37] LaFromboise T, Coleman HLK, Gerton J (1993). Psychological impact of biculturalism: evidence and theory. Psychol Bull.

[CR38] Lee JY, Kearns RA, Friesen W (2010). Seeking affective health care: Korean immigrants’ use of homeland medical services. Health Place.

[CR39] Levitt P, Glick Schiller N (2004). Conceptualizing simultaneity: a transnational social field perspective on society. Int Migr Rev.

[CR40] Lynd HM (1958). On shame and the search for identity.

[CR42] Neto F (2002). Loneliness and acculturation among adolescents from immigrant families in Portugal. J Appl Soc Psychol.

[CR43] Phinney JS, Horenczyk G, Liebkind K, Vedder P (2001). Ethnic identity, immigration, and well-being: an interactional perspective. J Soc Issues.

[CR44] Pinquart M, Sörensen S (2001). Influences on loneliness in older adults: a meta-analysis. Basic Appl Soc Psych.

[CR45] Prieto-Flores ME, Fernandez-Mayoralas G, Forjaz MJ, Rojo-Perez F, Martinez-Martin P (2011). Residential satisfaction, sense of belonging and loneliness among older adults living in the community and in care facilities. Health Place.

[CR46] Reijneveld SA (1998). Reported health, lifestyles, and use of health care of first generation immigrants in the Netherlands: do socioeconomic factors explain their adverse position?. J Epidemiol Community Health.

[CR47] Rubinstein RI, Parmelee PA, Altman I, Low SM (1992). Attachment to place and the representation of the life course by the elderly. Place attachment.

[CR48] Rudmin FW (2003). Critical history of the acculturation psychology of assimilation, separation, integration, and marginalization. Rev Gen Psychol.

[CR49] Sayad A (1999) Des illusions de l’émigré aux souffrances de l’immigré. In: Saada E (2000) Abdelmalek Sayad and the double absence. Toward a total sociology of immigration. French Polit Cult Soc 18(1):28–47. doi:10.3167/153763700782378193

[CR50] Schwartz SJ, Zamboanga BL (2008). Testing Berry’s model of acculturation: a confirmatory latent class approach. Cultur Divers Ethnic Minor Psychol.

[CR51] Silveira E, Allebeck P (2001). Migration, ageing and mental health: an ethnographic study on perceptions of life satisfaction, anxiety and depression in older Somali men in East London. Int J Soc Welf.

[CR52] Sonn CC, Fisher AT, Sonn CC, Bishop BJ (2002). Immigrant adaptation: Understanding the process through sense of community. Psychological sense of community: Research, applications and implications.

[CR53] Stronks K, Kulu-Glasgow I, Agyemang C (2009). The utility of “country of birth” for the classification of ethnic groups in health research: the Dutch experience. Ethn Health.

[CR54] Torres S, Phellas C (2013). Transnationalism and the study of aging and old age. Ageing in European societies.

[CR55] Treas J, Batlova J, Uhlenberg P (2009). Immigrants and aging. International handbook of population aging.

[CR56] Uysal-Bozkir Ö, Fokkema T, MacNeil-Vroomen JL, Van Tilburg TG, De Rooij SE (2015). Translation and validation of the De Jong Gierveld Loneliness Scale among older migrants living in the Netherlands. J Gerontol B Psychol Sci Soc Sci.

[CR57] Van Oudenhoven JP, Ward C, Masgoret AM (2006). Patterns of relations between immigrants and host societies. Int J Intercult Relat.

[CR58] Vedder P, Virta E (2005). Language, ethnic identity, and the adaptation of Turkish immigrant youth in the Netherlands and Sweden. Int J Intercult Relat.

[CR59] Verkuyten M (2004). The social psychology of ethnic identity.

[CR60] Victor C, Scambler SJ, Bowling A, Bond J (2005). The prevalence of, and risk factors for, loneliness in later life: a survey of older people in Great Britain. Ageing Soc.

[CR61] Victor C, Burholt V, Martin W (2012). Loneliness and ethnic minority elders in Great Britain: an exploratory study. J Cross Cult Gerontol.

[CR62] Vingerhoets AJJM, Van Tilburg MAL, Vingerhoets A (2005). The homesickness concept: Questions and doubts. Psychological aspects of geographical moves: homesickness and acculturation stress.

[CR63] Watt SE, Badger AJ (2009). Effects of social belonging on homesickness: an application of the belongingness hypothesis. Pers Soc Psychol Bull.

[CR64] Wiles JL, Allen RES, Palmer AJ, Hayman KJ, Keeling S, Kerse N (2009). Older people and their social spaces: a study of well-being and attachment to place in Aotearoa New Zealand. Soc Sci Med.

[CR65] Wu Z, Penning M (2013). Immigration and loneliness in later life. Ageing Soc.

[CR66] Yamada AM, Marsella AJ, Yamada SY (1997). The development of the ethnocultural identity behavioral index: psychometric properties and validation with Asian Americans and Pacific Islanders. Asian Am Pac Isl J Health.

[CR67] Yuval-Davis N (2006). Belonging and the politics of belonging. Patterns Prejudice.

